# Healthcare utilization patterns and medication burden in post-COVID syndrome: a cross-sectional analysis reveals four phenotypes

**DOI:** 10.3389/frhs.2026.1838358

**Published:** 2026-08-17

**Authors:** Jens Hamberger, Linda Kraus, Karin Meissner, Thilo Hinterberger, Thomas Loew, Oliver Schoeffski, Harald Gündel, Steffen Walter, Marc N. Jarczok, Katja Weimer

**Affiliations:** 1 Department of Psychosomatic Medicine, University Clinic Regensburg, Regensburg, Germany; 2 Department of Applied Natural Sciences and Health, Coburg University of Applied Sciences and Arts, Coburg, Germany; 3 Department of Psychosomatic Medicine and Psychotherapy, Ulm University Medical Center, Ulm, Germany; 4 School of Business and Economics, Chair of Health Care Management, Friedrich-Alexander University of Erlangen-Nuremberg, Nuremberg, Germany

**Keywords:** healthcare costs, healthcare utilization, long Covid, medication burden, patient clustering, polypharmacy, post-COVID syndrome, psychosomatic medicine

## Abstract

Post-COVID syndrome has emerged as a major public health challenge, yet comprehensive characterization of healthcare utilization patterns and medication burden within single cohorts remains limited. This cross-sectional analysis examined baseline data from 76 patients (49 ± 12 years, 62% female) enrolled in a randomized trial of psychosomatic treatment interventions for functional post-COVID syndrome. Participants completed the Health Care Utilization Questionnaire (HCU) assessing physician visits, hospitalizations, alternative healing methods, and other healthcare services over the preceding 12 months. Medication and other preparation (MOP) use was systematically documented and classified using the Anatomical Therapeutic Chemical (ATC) classification system. K-means clustering identified distinct patient phenotypes, and correlations between MOP count and healthcare utilization were examined using Spearman correlation coefficients. Healthcare utilization was universal, with 100% of participants engaging with healthcare services and a mean of 43.5 contacts per year. Medication burden was substantial, with 85.5% taking at least one medication, a mean of 4.57 medications per patient, and polypharmacy (≥5 MOP, all types) present in 39.5% of the cohort (46.1% of patients with any MOP). K-means clustering identified four distinct patient phenotypes: Low Utilizers (64.5%, *n* = 49), Moderate-High Utilizers (27.6%, *n* = 21), High-Intensity Users (6.6%), and one outlier. High-Intensity Users demonstrated the most striking profile with 9.60 mean medications, 75.0 total healthcare contacts annually, and mean out-of-pocket costs of €2,881. MOP count showed significant positive correlations with total doctor visits (*ρ* = 0.471, *p* < 0.001), total healthcare contacts (*ρ* = 0.344, *p* = 0.002), and out-of-pocket costs (*ρ* = 0.375, *p* = 0.001). Post-COVID syndrome patients demonstrate high healthcare engagement, substantial medication burden, and meaningful heterogeneity in utilization patterns. The identification of distinct patient clusters and strong correlations between medication burden and healthcare utilization provide insights for developing targeted clinical management strategies. This study is based on a selected clinical cohort, and findings should be interpreted with caution regarding generalizability to the broader post-COVID population. These findings may inform clinical management strategies and health system planning for post-COVID patients.

## 1 Introduction

The COVID-19 pandemic has fundamentally reshaped global healthcare since late 2019. While initial efforts focused on managing acute infections, it became increasingly clear that many patients continued to struggle long after their initial recovery. Post-COVID-19 syndrome—also called Long COVID or post-acute sequelae of SARS-CoV-2 infection (PASC)—has now emerged as a major public health challenge (1–3). The scale of this problem continues to grow as more people are affected.

Defining post-COVID syndrome (PCS) has proven challenging. The condition is generally characterized by symptoms persisting at least three months after acute infection that cannot be explained by other diagnoses. But prevalence estimates vary widely—from 10% to 35% in outpatient populations (4). Some studies report that up to 87% of patients experience at least one lingering symptom 60 days after recovery (4), while others find that roughly 26% of individuals haven't fully recovered even six to eight months post-infection (5). Among these patients, 55% report persistent fatigue, 25% experience ongoing breathing difficulties, and 26% develop depressive symptoms (5). In Germany, post-COVID syndrome resulted in 29,808 inpatient cases during 2021, representing a prevalence of 5.5% (4). By 2023, the number of PCS-related hospitalizations had decreased to 17,209 cases (48.9 per 100,000 population), though diagnostic complexity and resource utilization remained substantial (6). These numbers underscore the magnitude and evolving nature of the problem.

What makes post-COVID syndrome particularly difficult to manage is its phenomenological heterogeneity. The condition affects multiple organ systems simultaneously (2, 5, 7). Patients present with diverse combinations of symptoms: debilitating fatigue, cognitive impairment (often described as “brain fog”), shortness of breath, chest pain, heart palpitations, persistent headaches, sleep disturbances, anxiety, depression, and widespread musculoskeletal pain. This symptom variability (8) has led researchers to employ clustering and phenotyping approaches to identify meaningful patient subgroups (3, 9). Importantly, distinguishing between actual organ damage and functional deconditioning has proven critical for appropriate management. A structured diagnostic approach examining 231 post-COVID patients found that while 34.6% showed suspicious findings requiring advanced testing, only 44.4% of those had confirmed organ damage—the remainder exhibited functional complaints without detectable structural abnormalities (10). This distinction between “organic” and “functional” long COVID has important implications for treatment planning.

The healthcare burden is substantial. Compared to pre-infection baselines and matched controls, Long COVID patients show dramatically increased healthcare utilization. Nearly all patients (97.3%) require general practitioner consultations during follow-up (11), and 40% need at least one COVID-19-related healthcare contact after the acute phase (5). Among those initially hospitalized, 10% require rehospitalization (5). Notably, healthcare utilization doesn't decline quickly—it peaks around the fourth month and often continues well beyond (2).

The economic impact is substantial. In Germany, direct healthcare costs for post-COVID syndrome inpatients reached €136.6 million in 2021—an average of €4,583 per case (4). The 2023 update showed total inpatient costs of €67.9 million, with an average length of stay of 8.3 days (6). French data show that high-intensity Long COVID patients incur mean annual costs of €2,881 (11), while U.S. studies similarly document substantial excess expenditures (12, 13). These figures likely underestimate the true burden, as they typically exclude indirect costs such as lost productivity and informal caregiving. The most common concomitant diagnoses in hospitalized patients include pneumonia, critical illness polyneuropathy, dyspnea, chronic fatigue syndrome, and pulmonary embolisms (4).

Medication management in post-COVID syndrome presents another layer of complexity. Polypharmacy—generally defined as concurrent use of five or more medications—is common in chronic conditions and linked to increased costs, adverse drug events, and worse outcomes (14). Polypharmacy has been identified as a key driver of drug-drug interactions and increased healthcare burden in outpatient populations (15). Potentially inappropriate medication use remains prevalent, particularly among older adults (16). Given the multi-system nature of post-COVID syndrome, patients often receive multiple medications targeting different symptom domains. While the relationship between medication burden and healthcare utilization is well-established in other chronic conditions (17), we know surprisingly little about specific polypharmacy patterns in post-COVID populations. Do these patients follow similar patterns to other chronic disease populations? Or does the unique nature of post-COVID syndrome create distinct medication use profiles?

Beyond conventional medicine, patients increasingly turn to complementary approaches. Traditional Chinese medicine (TCM), including acupuncture and Chinese herbal therapy, has shown promise in treating post-COVID symptoms. A retrospective study of 79 post-COVID patients treated with TCM reported significant improvements in fatigue, physical performance, and dyspnea after an average of 7 consultations, with physicians rating global symptom improvement at 62% (18). This suggests that integrative treatment approaches warrant further investigation.

Researchers have begun using clustering techniques to make sense of this heterogeneity. Several studies have successfully identified distinct patient phenotypes based on symptom profiles and healthcare utilization intensity (3, 9). Electronic health record-based approaches have also shown promise for identifying Long COVID patients (2). However, most clustering studies have focused primarily on symptoms rather than healthcare utilization patterns or medication burden. Prescribing patterns vary substantially across demographic groups, reflecting underlying heterogeneity in patient populations (19).

The psychosomatic medicine perspective offers valuable insights here. Post-COVID syndrome involves complex interactions between biological, psychological, and social factors that influence both symptom persistence and healthcare-seeking behavior. The high prevalence of neuropsychiatric symptoms alongside physical complaints suggests that purely biomedical models are insufficient (5, 20). Psychosomatic medicine emphasizes the bidirectional relationships between mental and physical health and advocates for multidisciplinary care approaches (21). This perspective is particularly relevant given emerging evidence—for instance, pediatric Long COVID patients show a 40% increased risk of starting antidepressants (1). Psychosomatic rehabilitation programs for post-COVID patients are now being developed and evaluated, with interventions targeting patient expectations and placebo effects showing promise for improving rehabilitation outcomes (22). Yet few studies have examined post-COVID healthcare utilization through this integrated lens.

Despite growing research attention, critical gaps remain. While multiple studies document elevated healthcare costs and visit frequencies (4, 6, 11–13), few have systematically examined the full spectrum of healthcare utilization across multiple categories within a single cohort. We don't know enough about how different types of healthcare services—from primary care visits to hospitalizations to alternative medicine—cluster together in individual patients. The relationship between polypharmacy and healthcare utilization intensity remains poorly understood in this population. And although clustering approaches have identified symptom-based phenotypes (3, 9), we lack clear evidence about whether distinct healthcare utilization phenotypes exist and how these might relate to medication burden.

This study addresses these gaps by comprehensively characterizing healthcare utilization patterns and medication burden in post-COVID syndrome patients enrolled in a randomized trial of psychosomatic treatment interventions. We had *six specific objectives*: first, to describe healthcare utilization across multiple categories including physician visits, hospitalizations, alternative healing methods, other healthcare services, and medical aids; second, to quantify associated out-of-pocket costs; third, to examine the prevalence and patterns of polypharmacy; fourth, to identify distinct patient clusters based on healthcare utilization profiles using data-driven clustering methods; fifth, to investigate relationships between medication count and various dimensions of healthcare utilization; and sixth, to characterize how these patterns differ across identified clusters. By integrating data on both healthcare utilization and medication use within a well-characterized clinical cohort, we aim to provide insights that can inform more targeted clinical management strategies and evidence-based health policy decisions for this growing patient population. We acknowledge that this cohort represents a selected clinical population, and findings may not be generalizable to the broader post-COVID population.

## 2 Methods

This cross-sectional analysis utilized baseline data from the experimental study “Psychosomatic treatment options for functional post-COVID syndrome: a randomized trial of open-label placebos and breathing exercises” (PoCo-PA study) which enrolled 76 patients experiencing persistent symptoms following a SARS-CoV-2 infection. The main results of this study have not yet been published. The study had received approval from the Ethics Committee of the University of Ulm (Ethics Application Nos. 485/21 and 309/22) and was registered in advance with the German Registry of Clinical Trials (DRKS00028488). The study was conducted in accordance with the Declaration of Helsinki, and all participants provided written informed consent prior to participating.

### 2.1 Sample

The study was intended for patients who had recovered from COVID-19 and were experiencing long-term symptoms without any underlying organic causes. Suitable patients were recruited through the post-COVID specialty clinics of the Departments of Internal Medicine and Psychosomatic Medicine and Psychotherapy at the Ulm University Medical Center, Germany, as well as through general study advertising. Study participants were required to have persistent symptoms following a COVID-19 infection that had occurred at least 3 months prior, be between the ages of 18 and 70, have sufficient German language skills to complete questionnaires, and, according to their own statements, have no known organic causes for the symptoms following a COVID-19 infection that had been identified by a physician. Exclusion criteria included an implanted pacemaker, known hyperventilation syndrome, lactose intolerance, pregnancy and breastfeeding, as well as concurrent participation in other studies.

All patients were able to continue ongoing treatments during the study and were advised to interrupt or discontinue them only after consulting their respective treating physician. Treatments and doctor visits were recorded using the Health Care Utilization Questionnaire (HCU) (23). The study-related interventions consisted of additional treatment options (add-ons) to treatment-as-usual (TAU).

To inquire about SARS-CoV-2 infections or COVID-19 illnesses, participants were asked to provide the dates of their positive COVID-19 tests (self-test, rapid test, or PCR) during their COVID-19 illnesses. They were also asked about the number of SARS-CoV-2 vaccinations they had received and the type of vaccine.

### 2.2 Study design

The study comprised two on-site assessment sessions and one online follow-up session. During both assessment sessions, participants completed a series of questionnaires on a computer via the Unipark online platform (https://ww2.unipark.com; Tivian XI GmbH, Cologne, Germany), had an electrocardiogram (ECG) recorded, and provided a blood sample. Following the conclusion of the preliminary session, the participants were subjected to randomization into one of the four experimental groups. However, the only salient factor for this data analysis is that the participants did not yet know which group they belonged to at the time they completed the questionnaire during the first session.

### 2.3 Assessment of health care utilization

The healthcare utilization data were collected using a slightly modified version of the Health Care Utilization Questionnaire (HCU) (23). In particular, the medical specialties were asked about in greater detail than in the original questionnaire, and the question regarding medications was expanded to include supplementary substances. Upon reaching this section of the questionnaire, study participants were instructed to notify the experimenter. In order to enhance the reliability of the collected data, the inquiries concerning medications and other preparations (MOP) were completed in conjunction with a student enrolled in an advanced semester of medical school (research assistant). The participants were also asked to bring their list of medications and, if necessary, the packaging of any supplements they were taking to the appointment. This was done to ensure that no medications and preparations were omitted and that the correct nomenclature of the medications was entered. The nomenclature, dosages, and dosing schedules of all medications and supplementary substances that they have ingested during the preceding 12 months were recorded.

Furthermore, the questionnaire captured six distinct domains of healthcare utilization, medication and other preparations use, and associated costs covered by patients themselves (“out-of-pocket costs”). All inquiries addressed the preceding 12 months. Participants were asked to report the frequency of visits to 22 distinct medical specialties, that were categorized into nine clinically meaningful provider categories for further analysis. Inpatient and partial hospitalization data captured the number of nights or days, respectively, spent in hospital departments.

Participants reported the number of uses and associated out-of-pocket costs for five alternative healing modalities: osteopathy, acupuncture, naturopathy, traditional Chinese medicine, and other methods. The utilization and costs for four healthcare service types was captured: physiotherapy, massage, medical foot care, and continuing education/training programs. Additionally, participants were asked about the acquisition of medical aids, such as inhalers, compression bandages, exercise balls, mattresses, and other devices, along with associated costs.

### 2.4 Data preparation

Missing values were replaced with 0 for utilization analyses, assuming non-utilization when data were absent. The assumption of non-utilization for missing data is most plausible for utilization variables but should be considered a potential source of bias. After excluding entries with missing MOP information, 347 MOP entries from 65 patients were retained for analysis. This represented 85.5% of the baseline cohort reporting at least one medication or preparation. All MOP were classified using two systems: an own classification by preparation type and the Anatomical Therapeutic Chemical (ATC) classification system, maintained by the World Health Organization Collaborating Centre for Drug Statistics Methodology (24).

We categorized all entries by preparation type into Type 1 “Conventional medications and herbal preparations, including prescription pharmaceuticals and standardized herbal remedies, and those we believed have been prescribed”, Type 2 “Vitamins, encompassing single-vitamin preparations and vitamin combinations”, and Type 3 “Minerals and other supplements, including mineral supplements, amino acids, and other nutritional preparations”. Furthermore, the ATC classification system was used to categorize medications by their primary therapeutic indication and anatomical site of action. The first level of the ATC hierarchy (anatomical main group) was used for this analysis. To ensure accurate consolidation of medications with the same active ingredient, ATC codes were normalized where appropriate. Specifically, ATC codes A11CC and A11CC01 were consolidated to A11CC, as both codes refer to vitamin D and analogs according to the WHO ATC classification system.

Polypharmacy was categorized as: No medications (0), Low (1–4 medications), Moderate (5–9 medications), and High (≥10 medications) (14). For the primary analysis, polypharmacy was defined based on prescription medications and herbal preparations (Type 1) only. A secondary analysis examined polypharmacy based on total MOP count (Types 1, 2, and 3).

### 2.5 Statistics

Continuous variables were summarized using means, standard deviations, medians, interquartile ranges, and ranges. Categorical variables were presented as frequencies and percentages. Distributions were assessed visually using histograms and box plots.

K-means clustering was performed to identify distinct patient subgroups based on healthcare utilization patterns. Seven key variables were included: total doctor visits, total inpatient nights, total partial hospitalization days, total alternative healing uses, total other services, total medical aids, and total out-of-pocket costs. Variables were standardized using z-score transformation (mean = 0, SD = 1) prior to clustering. The optimal number of clusters (k = 4) was determined using the elbow method, examining within-cluster sum of squares across k = 2–10. The k-means algorithm utilized Lloyd's method with k-means++ initialization, Euclidean distance metric, convergence criterion of 0.0001, and maximum 300 iterations. Principal component analysis (PCA) was conducted for visualization of cluster separation, retaining two components explaining approximately 70% of variance.

K-means clustering was preferred over hierarchical clustering due to its scalability and suitability for identifying compact, well-separated clusters across the utilization variables. The elbow method was implemented by plotting within-cluster sum of squares (WCSS) against k values from 2 to 10; k = 4 was selected as the optimal solution based on the inflection point in the WCSS curve. Cluster stability was assessed by running the algorithm with 100 different random initializations and by performing a sensitivity analysis excluding the single extreme outlier patient and re-running the analysis with k = 3.

Spearman rank correlation coefficients were calculated to assess linear relationships between medication count and healthcare utilization variables. Statistical significance was assessed using two-tailed t-tests with significance levels set at *p* < 0.05. One-way analysis of variance (ANOVA) was performed to compare medication counts across patient clusters. *post-hoc* pairwise comparisons were conducted using Mann–Whitney *U*-tests with Bonferroni correction for multiple comparisons. The relationship between medication count and medication costs was assessed using Pearson correlation.

All analyses were conducted using SPSS 29.0 (IBM Corp., Armonk, NY) and Python 3.x with the following libraries: pandas (data management), numpy (numerical operations), scikit-learn (clustering and PCA), scipy (statistical tests), matplotlib and seaborn (visualization). Artificial intelligence assistance was provided by SciSpace AI (SciSpace, 2026) (25) for data integration and analysis workflow optimization, literature search, and language check.

## 3 Results

### 3.1 Sample description

The study population comprised 76 patients between 20 and 69 years with self-reported post-covid symptoms. One participant was accidentally given the second session's questionnaires during the baseline session. Consequently, the sociodemographic data, which were only collected during the first session, are missing for this participant, so the values do not add up to 100% (see Table 1). The majority of participants was in a relationship (80.3%), held at least a university entrance qualification (63.1%), and was employed either full-time or part-time (82.9%). It should be noted that all participants held German citizenship. Furthermore, 85.6% were enrolled in statutory health insurance. During the preceding 12-month period, the participants experienced an average absence from work of 124 days (SD = 141 days), although the median absence was 53 days (range from 0 to 365 days).

**Table 1:** Sociodemographic, health and COVID-19 related data of study participants.

Characteristic	All participants (*N* = 76)
Age (years)	49.3 ± 11.8
Sex (% female)	61.8%
Marital status	
Not married, with partner	9.2%
Married/in a registered partnership	71.7%
Divorced/separated	3.9%
Widowed	2.6%
Single (without partner)	11.8%
Educational attainment	
Lower secondary school	9.2%
Upper secondary school	22.4%
Technical college entrance qualification	18.4%
“Abitur” (university entrance qualification)	44.7%
Other	2.6%
None	1.3%
Employment status	
Full-time employed	50.0%
Part-time employed	32.9%
Part-time due to age	1.3%
Student	1.3%
Retiree or similar	6.6%
Unemployed	2.6%
Other	5.3%
Income	
0€ bis 1,500€	21.1%
1,501€ bis 3,000€	52.6%
>3,000€	26.2%
Health insurance	
Public health insurance	64.5%
Public health insurance and private supplemental insurance	21.1%
Subsidy and private health insurance (civil servant status)	6.6%
Private health insurance	6.6%
No information provided	1.3%
Absence from work (days)	124 ± 141
Months since first SARS-CoV-2 infection	21.5 ± 10.7
3-12 months	22.4%
13–24 months	40.8%
25–36 months	31.6%
> 36 months	3.9%
SARS-CoV-2 infections (number)	1.3 ± 0.6
one infection	77.6%
two infections	15.8%
three infections	6.6%
Vaccinations (number)	2.6 ± 0.8
zero vaccinations	1.3%
one vaccination	7.9%
two vaccinations	25.0%
three vaccinations	55.3%
four vaccinations	9.2%
Body mass index (kg/m^2^)	27.5 ± 5.7
between 18.5 to 24.9 (normal)	40.8%
25.0 to 29.9 (overweight)	34.2%
>30 (obesity)	25.0%
Blood pressure, systolic (mmHg)	130 ± 17
<120 mmHg (normal)	31.6%
120–139 mmHg (elevated)	40.8%
>139 mmHg (hypertension)	27.6%
Blood pressure, diastolic (mmHg)	86 ± 11
Heart rate (beats per minute)	72 ± 11

Sociodemographic data were missing for 1 participant (marital status, educational attainment, income, months since first SARS-CoV-2 infection, number of vaccinations).

In accordance with the inclusion criteria, all participants had had a SARS-CoV-2 infection more than 3 months prior to the commencement of the study; for 81.3% the infection had occurred at least 12 months earlier. Nearly 60% of participants had a body mass index (BMI) above the normal range (>25 kg/m^2^), and none had a BMI in the underweight range (<18.5 kg/m^2^). Furthermore, more than a quarter of the patients had blood pressure in the hypertensive range (systolic blood pressure ≥140 mmHg). The detailed sociodemographic and COVID-19 related data are presented in Table 1.

### 3.2 Doctor visits

All 76 patients (100%) reported at least four doctor visits in the 12 months prior to assessment, with a mean of 24.04 visits (SD = 16.66, range: 4–83). Table 2 presents healthcare utilization by provider category. Primary care was the most frequently utilized provider category, with 96.1% of patients averaging 8.25 visits (SD = 6.61) to their general practitioner. Other specialists [including gynecology, dermatology, ophthalmology, ear-nose-throat (ENT), radiology, and dentistry] were accessed by 92.1% of patients, with a mean of 5.57 visits (SD = 4.23). Nearly three-quarters of patients (72.4%) consulted internal medicine specialists, predominantly cardiology (51.3% of patients), reflecting cardiovascular complications associated with post-COVID syndrome. Over half of patients (56.6%) required neurological or psychiatric care, highlighting the substantial neuropsychiatric burden in this cohort. Notably, 35.5% of patients utilized alternative or complementary medicine providers, and 31.6% required pain management services including physiotherapy, indicating diverse treatment-seeking behaviors and ongoing symptom management needs in post-COVID patients (Table 2).

**Table 2:** Healthcare utilization by provider category (*N* = 76).

Rank	Category	Specialties (n)	Patients (%)	Total visits	Mean visits/patient (SD)
1	Primary Care	1 (general practitioner)	73 (96.1%)	627	8.25 (6.61)
2	Other Specialists	6 (gynecology, dermatology, ophtalmology, ENT, radiology, dentist)	70 (92.1%)	423	5.57 (4.23)
3	Internal Medicine Specialists	4 (cardiology, pulmonology, gastroenterology, internal medicine)	55 (72.4%)	168	2.21 (2.45)
4	Pain Management	2 (pain management/therapy, physiotherapy)	24 (31.6%)	168	2.21 (4.01)
5	Alternative/Complementary Medicine	3 (alternative practitioner, naturopathy, homeopathy)	27 (35.5%)	166	2.18 (4.32)
6	Neurological/Psychiatric	3 (neurology, psychiatry, psychosomatic medicine)	43 (56.6%)	165	2.17 (2.48)
7	Surgical Specialists	3 (surgery, orthopedics, urology)	39 (51.3%)	122	1.61 (2.12)
8	Hospital/Institutional	1 (hospital outpatient clinics)	19 (25.0%)	29	0.38 (0.70)
9	Occupational Health	2 (occupational physician, public health officer)	17 (22.4%)	22	0.29 (0.66)

ENT: Ear-nose-throat (otolaryngology). Patients may appear in multiple categories; total visits represents sum across all specialties.

### 3.3 Hospital-based care

One-third of participants (*n* = 25, 32.9%) required inpatient hospitalization in the 12-months period, accounting for 601 total hospital nights (mean: 24.04 nights among users, SD: 26.02, median: 14.0, range: 1–84 nights). Rehabilitation facilities accounted for 62.4% of all inpatient nights (375 nights, 16 patients), followed by psychosomatic medicine (22.1%, 133 nights, 7 patients), neurology (7.5%, 45 nights, 4 patients). While other departments (pulmonology, surgery, orthopedics, ENT, cardiology, gastroenterology) collectively represented 8.0% of inpatient nights. Partial/day hospitalization was utilized by only 3 patients (3.9%), primarily for psychosomatic care (67 of 77 days, 87.0%), and rehabilitation representing 13.0% (10 days). Overall, patients averaged 8.91 total hospital days per year (SD: 16.93, range: 0–84), with 26 patients (34.2%) experiencing at least one day of hospital-based care.

### 3.4 Alternative healing methods

Thirty patients (39.5%) utilized alternative healing methods, with 245 total uses and €18,616 in out-of-pocket costs. Among users, the mean number of uses was 8.17 (SD: 11.88, median: 4.0, range: 1–60), and mean costs were €620.53 (SD: €1,073.71, median: €200.00, range: €0-€4,800). Osteopathy was the most common modality, utilized by 21 patients (27.6%) with 113 total uses (46.1% of all alternative healing uses) and €8,100 in costs. Acupuncture was used by 10 patients (13.2%) with 60 uses and €3,600 in costs. Naturopathy, traditional Chinese medicine, and other methods were utilized by smaller proportions of patients.

### 3.5 Other health care services

Forty-six patients (60.5%) accessed other healthcare services, totaling 1,171 uses and €17,336 in costs. Physiotherapy dominated this category, accounting for 72.8% of all uses (853 uses, 33 patients, 43.4%) and 60.5% of costs (€10,488). Among physiotherapy users, the mean number of sessions was 25.85 (SD: 37.09, median: 12.0, range: 1–180). Massage therapy was utilized by 13 patients (17.1%) with 178 uses and €4,220 in costs. Medical foot care and continuing education/training programs were utilized by smaller proportions of patients.

### 3.6 Medical aids and devices

Eleven patients (14.5%) acquired 28 medical aids, totaling €1,825 in costs. Inhalers were the most common device, acquired by 7 patients (9.2%) with 10 total acquisitions and €1,050 in costs. Compression bandages were acquired by 6 patients (7.9%) with 11 acquisitions and €550 in costs. Exercise balls, mattresses, and other devices were acquired by smaller numbers of patients.

### 3.7 Medications and preparations use

Of the 76 patients, 85.5% reported taking at least one medication or preparation (MOP), while 11 patients (14.5%) reported no MOP use at baseline. The 65 patients with MOP data contributed a total of 347 medication/preparation entries, representing 312 unique MOP when accounting for variations in dosage, formulation, and brand names. When MOP were consolidated by active ingredient regardless of dosage variations, there are 256 unique active ingredients.

The mean number of MOP among users was 5.34 (SD = 3.70) per patient. The median number of MOP was 4.0, indicating a right-skewed distribution with some patients taking substantially more medications than the typical patient. The range extended from 1 to 18 MOP per patient, highlighting considerable heterogeneity in medication burden within the cohort.

#### 3.7.1 Most frequently reported medications and preparations by active ingredients

Table 3 presents the 30 most frequently reported MOP at baseline, consolidated by active ingredient to properly account for brand name variations and different dosage formulations of the same medication. This consolidation ensures that MOP with identical active ingredients and ATC codes (e.g., L-Thyroxin, L-Thyrox, and Euthyrox, all containing levothyroxine with ATC code H03AA01) are appropriately combined.

**Table 3:** Top 30 most frequently reported medications and preparations (MOP) by active ingredients.

Rank	Active ingredient	Patient count	Percentage of patients (*n* = 65)	ATC code	Type	Representative brand names/formulations (as named by the patient)
1	Vitamin D	14	21.5%	A11CC	2	Dekristol, Colecalciferol, Vitamin D 2,000 IE, Vitamin D3, Vigantoletten
2	Ibuprofen	13	20.0%	M01AE01	1	Ibu, Ibuprofen 400 mg, Ibuprofen 600 mg, Ibuflam
3	Levothyroxine	12	18.5%	H03AA01	1	L-Thyroxin, L-Thyrox, Euthyrox (various dosages)
4	Magnesium	11	16.9%	A12CC	3	Magnesium 400 mg, Magnesium 500 mg
5	Candesartan	10	15.4%	C09CA06	1	Candesartan 4 mg, 8 mg, 16 mg, 32 mg
6	Amlodipin	6	9.2%	C08CA01	1	Amlodipin 5 mg, Amlodipin 10mg
7	Ramipril	5	7.7%	C09AA05	1	Ramipril 2.5 mg, Ramipril 5mg
8	Pantoprazol	5	7.7%	A02BC02	1	Pantoprazol 20 mg, Pantoprazol 40mg
9	Metoprolol	4	6.2%	C07AB02	1	Metoprolol 23.75 mg, Metoprolol 47.5mg
10	Vitamin C	4	6.2%	A11GA01	2	Vitamin C 600 mg, Vitamin C 1000mg
11	Metamizole	3	4.6%	N02BB02	1	Novalgin, Novaminsulfon 500mg
12	Amitriptylin	3	4.6%	N06AA09	1	Amitriptylin 10 mg
13	Sertralin	3	4.6%	N06AB06	1	Sertralin 50 mg, 100 mg, 200 mg
14	Rivaroxaban	3	4.6%	B01AF01	1	Xarelto 2.5 mg, Xarelto 20 mg
15	Naproxen	3	4.6%	M01AE02	1	Naproxen 500 mg
16	Venlafaxin	2	3.1%	N06AX16	1	Venlafaxin 75 mg, Venlafaxin 150 mg
17	Probiotika	2	3.1%	—	3	Various probiotic preparations
18	Omega-3 Fatty Acids	2	3.1%	C10AX06	3	Omega 3 FS 1,400 mg
19	Nebivolol	2	3.1%	C07AB12	1	Nebivolol 5 mg
20	Selenium	2	3.1%	A12CE	1	Selen 200 mg, Selen 1,000 mg
21	Amino Acids	2	3.1%	—	3	Various amino acid supplements
22	Acetylsalicylic Acid	2	3.1%	B01AC06	1	ASS 100 mg
23	Clopidogrel	2	3.1%	B01AC04	1	Clopidogrel 75 mg
24	Atorvastatin	2	3.1%	C10AA05	1	Atorvastatin 40 mg, Atorvastatin 60 mg
25	St. John's Wort	2	3.1%	N06AP01	1	Johanniskraut
26	Zinc	2	3.1%	A12CB	3	Zink 15 mg, Zinkorot
27	Vitamin B12	2	3.1%	B03BA	2	Various B12 formulations
28	Salbutamol	2	3.1%	R03AC02	1	Sultanol, Salbuhexal
29	Bisoprolol	1	1.5%	C07AB07	1	Bisoprolol 5 mg
30	B-Complex	1	1.5%	V06	2	B-Komplex

Type 1 = Conventional medications and herbal preparations, that can be prescribed; Type 2 = Vitamins; Type 3 = Minerals and other supplements.

Vitamin D was the most commonly used supplement, reported by 14 patients (21.5% of patients with MOP data), appearing under various brand names and formulations. Ibuprofen was the second most prevalent medication, used by 13 patients (20.0%), appearing under various brand names and dosage forms. Levothyroxine was reported by 12 patients (18.5%), followed by magnesium supplements reported by 11 patients (16.9%). The cardiovascular medication candesartan was used by 10 patients (15.4%) across multiple dosage strengths.

#### 3.7.2 Classification by preparation type

Analysis of the classification revealed distinct patterns in the types of preparations utilized by post-COVID patients at baseline (column “Type” in Table 3). Conventional medications and herbal preparations (Type 1) constituted the majority of entries, accounting for 198 MOP (57.1% of all entries). This category encompassed prescription pharmaceuticals across multiple therapeutic classes (also see ATC classification), as well as standardized herbal preparations such as St. John's wort (Johanniskraut). Minerals and other supplements (Type 3) represented the second-largest category with 101 entries (29.1%), such as magnesium preparations in various formulations and dosages, but also included zinc, selenium, amino acids, omega-3 fatty acids, coenzyme Q10, and probiotics. Vitamins (Type 2) accounted for 37 entries (10.7%), with vitamin D preparations being most prevalent, followed by vitamin B12, vitamin C, and B-complex formulations. A small number of entries (*n* = 11, 3.2%) could not be classified due to missing or ambiguous preparation type information.

#### 3.7.3 ATC classification analysis

Medications with valid ATC codes (*n* = 281, 81.0% of all entries) were categorized according to their anatomical main group (Table 4). The three most prevalent ATC categories were alimentary tract and metabolism (A), cardiovascular system (C), and nervous system (N), collectively accounting for 59.1% of classified medications.

**Table 4:** Distribution of medications by ATC anatomical main group.

ATC code	Anatomical main group	Count	Percentage of classified medications
A	Alimentary tract and metabolism	62	22.1%
C	Cardiovascular system	58	20.6%
N	Nervous system	46	16.4%
R	Respiratory system	26	9.3%
H	Systemic hormonal preparations, excl. sex hormones and insulins	23	8.2%
M	Musculo-skeletal system	23	8.2%
B	Blood and blood forming organs	19	6.8%
V	Various	10	3.6%
G	Genito urinary system and sex hormones	7	2.5%
J	Antiinfectives for systemic use	3	1.1%
D	Dermatologicals	2	0.7%
L	Antineoplastic and immunomodulating agents	1	0.4%
S	Sensory organs	1	0.4%

Alimentary Tract and Metabolism (A): This category (*n* = 62, 22.1%) included proton pump inhibitors such as pantoprazol, vitamin and mineral supplements classified under ATC codes (e.g., vitamin D as A11CC, magnesium as A12CC), antidiabetic medications (metformin, ozempic), and various nutritional supplements. The prominence of this category reflects both gastrointestinal symptom management and nutritional supplementation strategies.

Cardiovascular System (C): Cardiovascular medications (*n* = 58, 20.6%) represented a substantial proportion of baseline medication use. This category encompassed calcium channel blockers (amlodipine, nifedipine, lercanidipine), angiotensin II receptor blockers (candesartan in multiple dosages), ACE inhibitors (ramipril), beta-blockers (bisoprolol, metoprolol, nebivolol, propranolol), diuretics (torasemid, HCT, indapamide), and lipid-modifying agents (atorvastatin, rosuvastatin, fenofibrate). The high prevalence of cardiovascular medications suggests either pre-existing cardiovascular comorbidities or cardiovascular sequelae of COVID-19 infection.

Nervous System (N): Medications targeting the nervous system (*n* = 46, 16.4%) included analgesics (ibuprofen, novaminsulfon, naproxen, paracetamol), antidepressants (sertralin, venlafaxin, citalopram, amitriptyline, mirtazapine), medications for neuropathic pain (pregabalin), antimigraine preparations (sumatriptan), and herbal psychotropic preparations (Johanniskraut/St. John's wort). This diverse array of nervous system medications reflects the neurological and neuropsychiatric manifestations commonly reported in post-COVID patients, including headache, neuropathic pain, depression, and anxiety.

Respiratory System (R): Respiratory medications (*n* = 26, 9.3%) included bronchodilators (salbutamol/sultanol) and combination inhalers containing corticosteroids and long-acting beta-agonists (Foster, Symbicort, Relvar). The presence of these medications indicates persistent respiratory symptoms or exacerbation of pre-existing respiratory conditions following COVID-19 infection.

Systemic Hormonal Preparations (H): This category (*n* = 23, 8.2%) was dominated by thyroid hormone replacement therapy. When properly consolidated by active ingredient, levothyroxine (appearing under brand names L-Thyroxin, L-Thyrox, and Euthyrox, all with ATC code H03AA01) was used by 12 patients (18.5%), making it the second most prevalent medication in the cohort. The high frequency of thyroid medications suggests either pre-existing thyroid disorders or potential thyroid dysfunction associated with post-COVID syndrome.

Musculo-skeletal System (M): Medications in this category (*n* = 23, 8.2%) primarily consisted of non-steroidal anti-inflammatory drugs (NSAIDs) such as ibuprofen, naproxen, and diclofenac, as well as medications for gout (allopurinol). The prevalence of these medications underscores the significance of musculoskeletal pain and inflammation in the post-COVID symptom complex.

Blood and Blood Forming Organs (B): This category (*n* = 19, 6.8%) included anticoagulants (xarelto/rivaroxaban, clopidogrel), antiplatelet agents (ASS/aspirin), and hematinics (iron preparations, vitamin B12, folic acid). The presence of anticoagulants may reflect either pre-existing cardiovascular risk factors or thrombotic complications associated with COVID-19.

The remaining ATC categories (V, G, J, D, L, S) collectively accounted for 8.5% of classified medications, representing more specialized therapeutic areas including antiinfectives (J), dermatological preparations (D), genitourinary medications (G), antineoplastic and immunomodulating agents (L), and preparations for the sensory organs (S).

#### 3.7.4 Polypharmacy prevalence and distribution

The distribution of patients across polypharmacy categories revealed a concerning prevalence of complex medication regimens. Only 23.1% of patients were taking minimal medications (1–2), while the majority (76.9%) were taking three or more concurrent medications. Notably, 46.1% of patients with MOP data (30/65) met criteria for polypharmacy (≥5 MOP of any type). Using the primary definition based on prescription medications only (Type 1), 13 patients (17.1%) met polypharmacy criteria (≥5 Type 1 MOPs). When including all MOP types, 30 patients (39.5%) met criteria (30/65 patients with MOP data=46.1%), with nearly one in eight (12.3%) patients experiencing excessive polypharmacy (≥10 medications).

**Figure 1:**
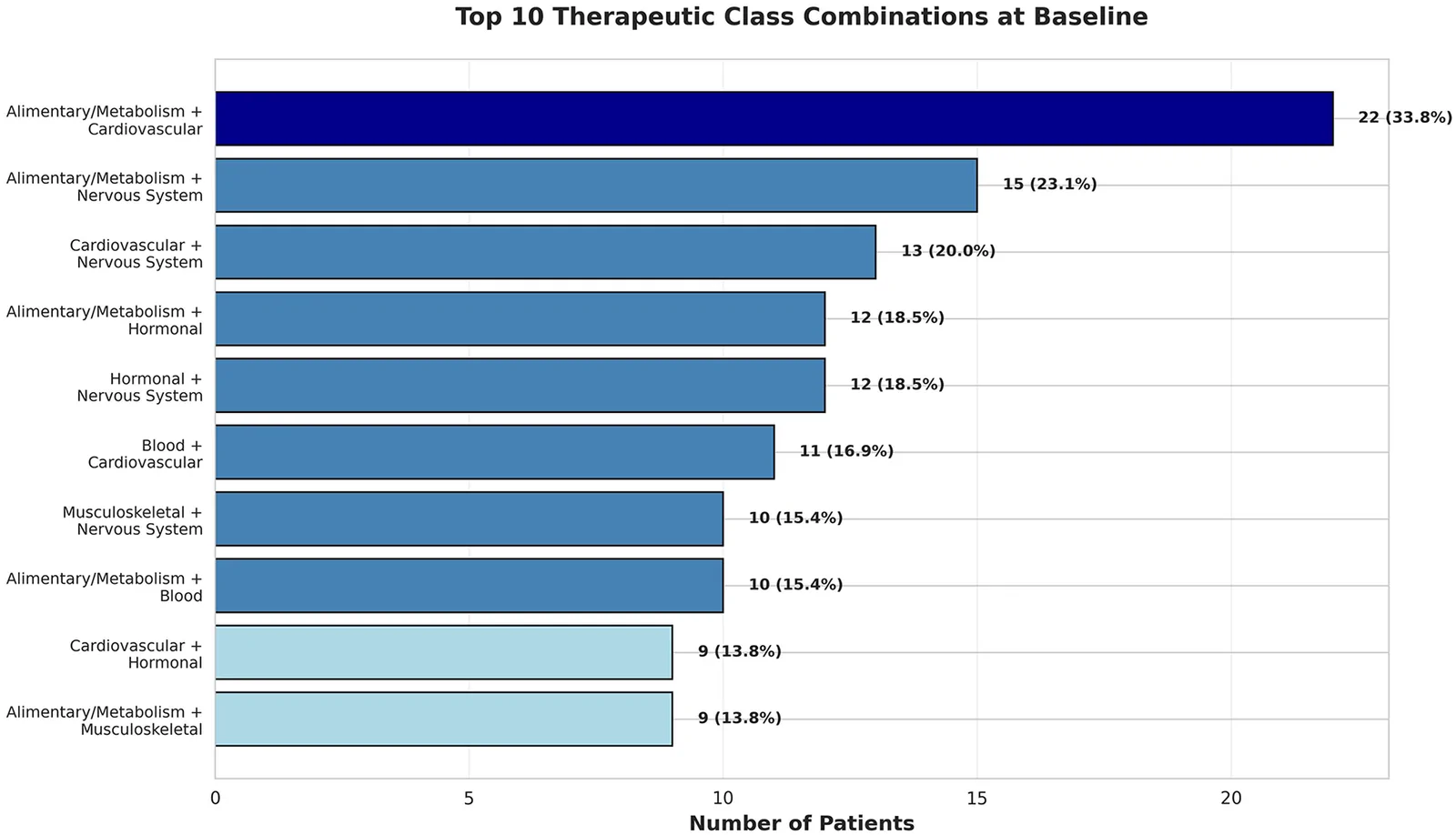
Top ten therapeutic class combinations. Most common two-class medication combinations among post-COVID syndrome patients (*n* = 65) who took any medication. Medications were classified using the Anatomical Therapeutic Chemical (ATC) classification system. Bar colors indicate frequency: dark blue (≥20 patients), middle blue (10-19 patients), light blue (<10 patients). The most prevalent combination was Alimentary/Metabolism+Cardiovascular medications (33.8% of patients), followed by Alimentary/Metabolism+Nervous System (23.1%), and Cardiovascular+Nervous System (20.0%).

Analysis of therapeutic class combinations using ATC first-level classification revealed patterns of multimorbidity and treatment complexity (Figure 1).

### 3.8 Patient clustering analysis

K-means clustering identified four distinct patient subgroups with markedly different healthcare utilization patterns (Table 5). Cluster 0 (Low Utilizers, *n* = 49, 64.5%) represented the majority of patients, characterized by minimal healthcare use with 14.4 doctor visits, 1.6 inpatient nights, and €193 annual out-of-pocket costs. Cluster 1 (High-Intensity Users, *n* = 5, 6.6%) demonstrated intensive utilization across multiple domains, with 49.6 doctor visits, 27.4 inpatient nights, 16.8 partial hospitalization days, and €2,881 annual costs. Cluster 2 (Moderate-High Utilizers, *n* = 21, 27.6%) showed substantial outpatient care utilization with 40.5 doctor visits and €651 annual costs, but minimal hospitalization. Cluster 3 (Extreme Outlier, *n* = 1, 1.3%) represented a single patient with exceptional utilization (83 doctor visits, 42 inpatient nights, 42 partial hospitalization days).

**Table 5:** Patient cluster characteristics (*N* = 76).

Characteristic	Cluster 0: Low Utilizers (*n* = 49, 64.5%)	Cluster 1: High-Intensity Users (*n* = 5, 6.6%)	Cluster 2: Moderate-High Utilizers (*n* = 21, 27.6%)	Cluster 3: Extreme Outlier (*n* = 1, 1.3%)
Doctor Visits				
Mean (SD)	14.4 (7.5)	49.6 (8.0)	40.5 (11.3)	83.0 (-)
Median (Range)	13.0 (0–30)	49.0 (38–55)	38.0 (27–67)	83.0 (83–83)
Inpatient Nights				
Mean (SD)	1.6 (5.6)	27.4 (15.5)	0.9 (3.0)	42.0 (-)
Median (Range)	0.0 (0–31)	30.0 (1–42)	0.0 (0–12)	42.0 (42–42)
Partial Hospitalization Days				
Mean (SD)	0.2 (1.6)	16.8 (18.8)	0.5 (2.2)	42.0 (-)
Median (Range)	0.0 (0–10)	10.0 (0–42)	0.0 (0–10)	42.0 (42–42)
Alternative Healing Uses				
Mean (SD)	1.6 (4.5)	10.2 (13.7)	2.4 (4.3)	0.0 (-)
Median (Range)	0.0 (0–30)	4.0 (0–30)	0.0 (0–12)	0.0 (0–0)
Other Services				
Mean (SD)	5.1 (12.0)	25.4 (15.8)	32.8 (40.5)	40.0 (-)
Median (Range)	0.0 (0–80)	30.0 (6–40)	12.0 (0–140)	40.0 (40–40)
Medical Aids				
Mean (SD)	0.2 (0.6)	0.6 (0.9)	0.4 (0.9)	0.0 (-)
Median (Range)	0.0 (0–4)	0.0 (0–2)	0.0 (0–4)	0.0 (0–0)
Out-of-Pocket Costs (€)				
Mean (SD)	193 (353)	2,881 (1,545)	651 (1,006)	240 (-)
Median (Range)	54 (0–1,350)	3,293 (860–4,800)	300 (0–3,600)	240 (240–240)
Total Healthcare Contacts				
Mean (SD)	19.5 (14.5)	75.0 (19.5)	73.3 (42.8)	123.0 (-)
Median (Range)	16.0 (0–80)	68.0 (55–111)	61.0 (29–179)	123.0 (123–123)
Total Hospital Days				
Mean (SD)	1.8 (6.4)	44.2 (24.0)	1.4 (3.5)	84.0 (-)
Median (Range)	0.0 (0–31)	35.0 (1–84)	0.0 (0–12)	84.0 (84–84)
HCU Categories Used				
Mean (SD)	1.9 (0.9)	4.4 (0.9)	3.3 (1.0)	4.0 (-)
Median (Range)	2.0 (0–4)	4.0 (4–6)	3.0 (2–6)	4.0 (4–4)

Sensitivity analyses confirmed moderate cluster stability. The silhouette score for the k = 4 solution was 0.41, indicating moderate cluster separation. Removal of the extreme outlier patient (Cluster 3, *n* = 1) and re-analysis with k = 3 yielded cluster patterns broadly consistent with the Low Utilizers, High-Intensity Users, and Moderate-High Utilizers of the main solution, supporting the robustness of the core findings.

### 3.9 Medication – healthcare utilization relationship

MOP count demonstrated significant positive correlations with multiple healthcare utilization variables. The strongest correlation was observed with total doctor visits (Spearman *ρ* = 0.471, *p* < 0.001), indicating that patients taking more medications had substantially higher physician visit frequencies. Significant positive correlations were also found with total healthcare contacts (*ρ* = 0.344, *p* = 0.002), and total out-of-pocket costs (*ρ* = 0.375, *p* = 0.001). No significant correlations were observed between MOP count and total medical aids, total alternative healing uses, inpatient nights, partial hospitalization days, other services, or total hospital days.

One-way ANOVA revealed significant differences in MOP counts across patient clusters (F = 7.39, *p* = 0.001). *post-hoc* Mann–Whitney U tests with Bonferroni correction demonstrated that Cluster 1 (High-Intensity Users) had significantly higher medication counts (mean=9.60, SD = 2.30) compared to Cluster 0 (Low Utilizers; mean = 3.61, SD = 3.40; *p* = 0.001) and Cluster 2 (Moderate-High Utilizers; mean = 5.62, SD = 4.32; *p* = 0.033).

To further explore the relationship between pharmaceutical use and healthcare utilization, we conducted separate correlation analyses for medications (Type 1: prescription drugs) and other preparations (Types 2 & 3: vitamins, minerals, and dietary supplements). Interestingly, other preparations showed a significant positive correlation with total doctor visits (*ρ* = 0.363, *p* = 0.001), while medications showed no significant correlations with any healthcare utilization variable (all *p* > 0.05). Neither medication nor supplement use correlated significantly with inpatient hospitalization nights or partial hospitalization days (all *p* > 0.05). Alternative healing services, other services, and medical aids showed insufficient variance for correlation analysis, as most patients reported zero use of these services at baseline.

The ANOVA for medications only (Type 1) revealed no differences in medication counts across patient clusters (F = 0.52, *p* = 0.672). In contrast, the ANOVA for other preparations (Types 2 & 3) showed significant differences in the preparation counts between patient clusters (F = 10.95, *p* < 0.001). *post-hoc* Mann–Whitney U tests with Bonferroni correction demonstrated that Cluster 0 had significantly lower other preparation counts (mean = 1.16, SD = 1.58) compared to Cluster 1 (mean = 6.60, SD = 1.02; *p* < 0.001).

## 4 Discussion

### 4.1 Summary of main findings

This cross-sectional analysis of 76 post-COVID syndrome patients from the PoCo-PA study provides comprehensive insights into healthcare utilization patterns and medication burden in this emerging patient population. Our findings reveal high healthcare engagement, with 100% of participants utilizing healthcare services and a mean of 43.5 healthcare contacts per year. The medication burden was substantial, with 85.5% of patients taking at least one medication and a mean of 4.57 medications per patient. Polypharmacy (≥5 MOP, all types) was present in 39.5% of all patients (46.1% of patients with any MOP).

Using data-driven clustering methods, we identified four distinct patient phenotypes. Cluster 1 (High-Intensity Users, 6.6% of the sample) demonstrated the most striking profile with a mean of 9.60 medications, 75.0 total healthcare contacts annually, and mean out-of-pocket costs of €2,881. In contrast, Cluster 0 (Low Utilizers, 64.5%) showed considerably lower utilization with 3.61 medications and 19.5 healthcare contacts. We observed significant positive correlations between medication count and multiple dimensions of healthcare utilization, including total doctor visits (*ρ* = 0.471, *p* < 0.001), total healthcare contacts (*ρ* = 0.344, *p* = 0.002), and out-of-pocket costs (*ρ* = 0.375, *p* = 0.001), suggesting that medication burden serves as a meaningful marker of overall healthcare system engagement.

### 4.2 Healthcare utilization

Our finding of universal healthcare utilization aligns closely with recent population-based studies in other countries. Yang and colleagues reported that 97.3% of community-managed Long COVID adults in France had general practitioner consultations during follow-up (11), nearly identical to our 100% utilization rate. Mu and colleagues, analyzing 282,080 individuals with Long COVID over two years, documented persistently elevated healthcare utilization compared to matched controls (9), with temporal patterns consistent with the chronic nature of symptoms in our cohort.

The out-of-pocket costs documented in our study provide important context for understanding the economic burden. Our mean cost of €651 across all clusters, with High-Intensity Users incurring €2,881 annually, aligns with French data (11). These figures are particularly striking given that Germany and France both have comprehensive health insurance systems, suggesting that even in well-resourced healthcare contexts, patients face substantial direct financial burdens (12, 13).

The inpatient utilization patterns warrant particular attention. While the majority (64.5% in Cluster 0) had no hospitalizations, a subset experienced substantial inpatient care, with Cluster 1 averaging 44.2 hospital days. These findings resonate with German nationwide inpatient data reported by Walter and colleagues, who documented 29,808 post-COVID syndrome inpatients in 2021 with a mean length of stay of 11.5 days and total direct healthcare costs of €136.6 million (4). Their updated 2023 analysis showed 17,209 PCS-related hospitalizations with an average length of stay of 8.3 days and total costs of €67.9 million (6), indicating that while absolute numbers decreased, diagnostic complexity and resource intensity remained high.

### 4.3 Medication burden and polypharmacy

The prevalence of polypharmacy in our cohort (39.5%) represents a novel contribution to the post-COVID literature, as few studies have systematically examined medication burden in this population. The mean medication count of 4.57 suggests that post-COVID syndrome patients are being managed with multi-drug regimens comparable to those used in other chronic disease populations, with medications spanning diverse therapeutic classes reflecting the multi-system symptomatology characteristic of post-COVID syndrome.

The strong positive correlation between medication count and doctor visits (*ρ* = 0.471, *p* < 0.001) suggests that patients with more complex symptom profiles may require more medications and consequently need more frequent medical monitoring. To further explore this relationship, we conducted separate correlation analyses for prescription medications (Type 1) and other preparations (Types 2 & 3: vitamins, minerals, and dietary supplements). Interestingly, other preparations showed a significant positive correlation with total doctor visits (Spearman *ρ* = 0.363, *p* = 0.001), while prescription medications showed no significant correlations with any healthcare utilization variable (all *p* > 0.05). This pattern may tentatively suggest—as an exploratory hypothesis—that supplement use could serve as a marker of health-seeking behavior and proactive engagement with the healthcare system, rather than reflecting disease burden *per se*. This interpretation is speculative given the cross-sectional study design and should be tested in future prospective research. In contrast, prescription medication use—which typically reflects underlying disease burden—did not correlate with healthcare utilization in our cohort, possibly because many medications in post-COVID patients are for stable chronic conditions (e.g., antihypertensives, thyroid medications) that do not directly drive healthcare utilization. The correlation between medication count and out-of-pocket costs (*ρ* = 0.375, *p* = 0.001) highlights the financial implications of polypharmacy, particularly relevant given that many post-COVID patients experience reduced work capacity. Tran and colleagues documented a 40% increased risk of antidepressant initiation among children and adolescents with Long COVID (1), suggesting that psychotropic medication use represents an important component of the medication burden across age groups.

Kraft and colleagues reported significant improvements in post-COVID symptoms following TCM treatment, with physicians rating global symptom improvement at 62% after an average of seven consultations (18). The most common TCM treatments included acupuncture (85% of patients) and Chinese pharmacological therapy (77%), with significant alleviation observed in fatigue, physical performance, and dyspnea.

### 4.4 Utilization patterns

Our identification of four distinct patient clusters extends previous clustering work that has primarily focused on symptom profiles (3). The High-Intensity Users (Cluster 1) are of particular clinical and policy interest. Representing only 6.6% of our cohort but accounting for disproportionate healthcare resources, these patients had significantly higher medication counts (mean 9.60 vs. 3.61 in Low Utilizers, *p* = 0.001) and substantially elevated costs. Hong and colleagues documented elevated healthcare utilization that peaked at and continued beyond the fourth month following infection (2), suggesting that identifying high-intensity utilizers might support more targeted interventions, though prospective studies are needed to confirm this.

The distinction between patients with organic pathology vs. functional complaints, as articulated by our co-author Kersten and colleagues, provides important context for understanding this heterogeneity (10). In their structured diagnostic evaluation of 231 post-COVID patients, they found that while 34.6% showed suspicious findings requiring advanced testing, only 44.4% of those had confirmed organ damage—the remainder exhibited functional complaints without detectable structural abnormalities. This distinction has profound implications for treatment planning and may partially explain the variability in healthcare utilization patterns we observed.

### 4.5 Clinical implications

The findings have several important implications for clinical practice. First, the universal healthcare utilization and substantial medication burden underscore the need for coordinated, multidisciplinary care approaches. The fragmentation of care across multiple providers creates risks for medication interactions, duplicative testing, and inconsistent treatment plans. Establishing dedicated post-COVID clinics with integrated care teams represents a promising approach to addressing this coordination challenge.

Second, the strong correlation between medication count and healthcare utilization suggests that medication burden could serve as a readily assessable marker for identifying patients who may benefit from intensified care coordination. Systematic medication reviews, particularly for patients taking five or more medications, could help identify opportunities for deprescribing, address potential drug interactions, and optimize therapeutic regimens. Clinical decision support systems have been shown to improve prescribing safety and reduce inappropriate medication use in similar outpatient settings (26).

Third, the identification of distinct patient clusters suggests that one-size-fits-all approaches are unlikely to be optimal. Low Utilizers may benefit from supportive self-management strategies and periodic monitoring, while High-Intensity Users may benefit from more intensive care coordination. Developing risk stratification tools that incorporate medication burden, healthcare utilization patterns, and symptom profiles could enable more personalized care planning.

From a psychosomatic medicine perspective, our findings reinforce the importance of addressing the complex interplay between biological, psychological, and social factors. The substantial use of psychotropic medications highlights the need for integrated mental health support as a core component of post-COVID care. Wedekind and colleagues are currently evaluating expectation-focused interventions designed to optimize rehabilitation outcomes in post-COVID patients (22), recognizing that patient expectations and placebo effects represent important therapeutic mechanisms.

The distinction between organic pathology and functional deconditioning, as emphasized by Kersten and colleagues (10), has direct implications for treatment selection. Patients with confirmed organ damage require targeted medical interventions addressing specific pathophysiology, while those with functional symptoms may benefit more from graded exercise therapy, cognitive-behavioral approaches, and psychosomatic rehabilitation.

### 4.6 Healthcare policy implications

The findings have significant implications for healthcare policy and resource allocation. First, the substantial healthcare utilization and costs documented in our cohort and corroborated by large-scale population studies underscore the need for dedicated funding streams and reimbursement structures for post-COVID care. The German nationwide data showing €136.6 million in direct inpatient costs in 2021 (4) and €67.9 million in 2023 (6) represent only the inpatient component of the total burden.

Second, the concentration of resource utilization among High-Intensity Users suggests that targeted case management programs could yield substantial returns on investment. If 6.6% of patients account for a disproportionate share of costs, then intensive care coordination for this subgroup could potentially reduce overall system costs while improving patient outcomes.

Third, the medication burden has implications for pharmaceutical policy and prescribing practices. Reimbursement models that compensate providers for comprehensive medication reviews and care coordination activities could better support high-quality post-COVID care. Outpatient prescribing patterns often reflect broader health system challenges and may require targeted policy interventions (27).

Fourth, the persistent nature of healthcare utilization documented in longitudinal studies (2, 9) indicates that health systems must plan for sustained demand. The decrease in PCS-related hospitalizations from 29,808 in 2021 to 17,209 in 2023 (4, 6) suggests that the acute surge may be subsiding, but the continued diagnostic complexity indicates that specialized expertise will remain necessary.

Fifth, the out-of-pocket costs—ranging from €193 for Low Utilizers to €2,881 for High-Intensity Users—raise equity concerns. Patients with reduced work capacity face the dual burden of decreased income and increased healthcare expenses. Policy interventions to reduce financial barriers to care could improve access and reduce disparities.

### 4.7 Study limitations

Several limitations warrant acknowledgment. First, the sample was recruited from specialized post-COVID clinics and through study advertising, representing a highly selected clinical cohort. This likely favored patients with more severe and persistent symptoms, substantially limiting generalizability to the broader post-COVID population. Population-based studies would provide important complementary evidence. Second, the cross-sectional design precludes causal inference regarding the relationships between medication burden and healthcare utilization. Longitudinal studies would provide more definitive insights. Third, the sample size of 76 patients limited statistical power for some cluster comparisons, particularly for Cluster 3 which contained only one patient. Given the small sample size and the single extreme outlier in Cluster 3 (*n* = 1), these cluster solutions should be interpreted as preliminary patterns requiring replication in larger cohorts. Fourth, our assessment of healthcare utilization relied on self-report, which is subject to recall bias, particularly for infrequent events occurring early in the 12-month recall window. Patients with more severe symptoms may recall their utilization more readily, potentially introducing differential recall bias. Administrative claims data would provide more objective capture of healthcare encounters. We also lacked detailed information about pre-existing conditions prior to the COVID-19 infection. Additionally, we replaced missing values with 0 for utilization data and did not perform multiple imputation sensitivity analyses as they are beyond the scope of this exploratory study. Fifth, the study was conducted in Germany within a specific healthcare system context, which may limit generalizability to other countries. We also did not systematically assess the appropriateness of medications or healthcare utilization. Furthermore, analyses were limited to bivariate correlations and descriptive cluster comparisons. Key confounders such as age, sex, BMI, comorbidities, and disease severity were not adjusted for, as multivariable analysis was not feasible given the sample size. This represents a prominent limitation for interpreting the reported associations and future studies with larger samples should employ multivariable approaches.

### 4.8 Future research directions

The findings identify several important directions for future research. First, longitudinal studies tracking healthcare utilization and medication burden over extended follow-up periods are needed to understand trajectories of care engagement and identify predictors of persistent high utilization. Second, intervention studies testing care coordination models, medication optimization programs, and integrated care approaches are critically needed. Randomized controlled trials comparing integrated multidisciplinary care to usual care would provide evidence. Third, research examining the appropriateness of medications and healthcare services in post-COVID populations is essential to identify opportunities to optimize care quality while potentially reducing costs. Fourth, comparative effectiveness research examining different care models for post-COVID syndrome is needed to inform health system planning. Fifth, research on the role of psychosomatic and rehabilitative interventions in reducing medication burden represents an important gap. The expectation-focused interventions being investigated by Wedekind and colleagues (22) represent one promising approach that warrants rigorous evaluation. Sixth, health economic analyses examining the cost-effectiveness of different management strategies are essential. Cost-effectiveness analyses incorporating both direct healthcare costs and indirect costs such as lost productivity would provide crucial evidence for policy decisions. Finally, as the pandemic evolves, research tracking changes in post-COVID syndrome epidemiology and healthcare utilization patterns over time will be essential. The decrease in hospitalizations documented between 2021 and 2023 (4, 6) may reflect changing viral characteristics, population immunity, or improved acute care, but the implications for long-term care needs remain uncertain.

### 4.9 Conclusion

This comprehensive analysis of healthcare utilization and medication burden in post-COVID syndrome patients reveals substantial engagement with the healthcare system, significant polypharmacy, and meaningful heterogeneity in utilization patterns. The identification of distinct patient clusters and the strong correlations between medication burden and healthcare utilization provide insights that can inform more targeted clinical management strategies and evidence-based health policy decisions. From a psychosomatic medicine perspective, our findings underscore the importance of integrated care approaches that address the complex interplay of biological, psychological, and social factors in this emerging patient population. As the burden of post-COVID syndrome continues to evolve, ongoing research and adaptive health system responses will be essential to optimize outcomes while ensuring sustainable, equitable care delivery.

## Data Availability

The raw data supporting the conclusions of this article will be made available by the authors, without undue reservation.
